# Non-Obstructive Azoospermia and Intracytoplasmic Sperm Injection: Unveiling the Chances of Success and Possible Consequences for Offspring

**DOI:** 10.3390/jcm13164939

**Published:** 2024-08-21

**Authors:** Ahmad Majzoub, Marina C. Viana, Arnold P. P. Achermann, Isadora T. Ferreira, Rita J. Laursen, Peter Humaidan, Sandro C. Esteves

**Affiliations:** 1 Department of Urology, Hamad Medical Corporation, Doha 3050, Qatar; dr.amajzoub@gmail.com; 2 Department of Clinical Urology, Weill Cornell Medicine-Qatar, Doha 3050, Qatar; 3 ANDROFERT, Andrology & Human Reproduction Clinic, Campinas 13075-460, SP, Brazil; marinacorreaviana@gmail.com (M.C.V.); arnoldpp@gmail.com (A.P.P.A.); 4 Faculty of Medical Sciences, Pontifical Catholic University of Campinas, Campinas 13087-571, SP, Brazil; isadoraferreira7@gmail.com; 5 Skive Fertility Clinic, Skive Regional Hospital, 7800 Skive, Denmark; rita.laursen@midt.rm.dk (R.J.L.); peter.humaidan@midt.rm.dk (P.H.); 6 Department of Clinical Medicine, Faculty of Health, Aarhus University, 8000 Aarhus, Denmark; 7 Department of Surgery, Division of Urology, State University of Campinas (UNICAMP), Campinas 13083-887, SP, Brazil

**Keywords:** male infertility, non-obstructive azoospermia, spermatogenic failure, sperm retrieval, testis, intracytoplasmic sperm injection, assisted reproductive technology, pregnancy, offspring health, review

## Abstract

Non-obstructive azoospermia (NOA) is found in up to 15% of infertile men. While several causes for NOA have been identified, the exact etiology remains unknown in many patients. Advances in assisted reproductive technology, including intracytoplasmic sperm injection (ICSI) and testicular sperm retrieval, have provided hope for these patients. This review summarizes the chances of success with ICSI for NOA patients and examines preoperative factors and laboratory techniques associated with positive outcomes. Furthermore, we reviewed possible consequences for offspring by the use of ICSI with testicular sperm retrieved from NOA patients and the interventions that could potentially mitigate risks. Testicular sperm retrieved from NOA patients may exhibit increased chromosomal abnormalities, and although lower fertilization and pregnancy rates are reported in NOA patients compared to other forms of infertility, the available evidence does not suggest a significant increase in miscarriage rate, congenital malformation, or developmental delay in their offspring compared to the offspring of patients with less severe forms of infertility or the offspring of fertile men. However, due to limited data, NOA patients should receive specialized reproductive care and personalized management. Counseling of NOA patients is essential before initiating any fertility enhancement treatment not only to mitigate health risks associated with NOA but also to enhance the chances of successful outcomes and minimize possible risks to the offspring.

## 1. Introduction

Male infertility is a disease of the male reproductive system, caused primarily by congenital and genetic conditions; anatomical, endocrine, functional or immunological abnormalities of the reproductive system; genital tract infections; cancer and its related treatment; and sexual disorders incompatible with intercourse. Inadequate lifestyle, exposure to toxicants, and advanced paternal age are risk factors acting alone or exacerbating the impact of known causative factors [1,2,3,4].

Azoospermia, characterized by the absence of sperm in the ejaculate, is the most severe form of male infertility and it can be associated with one or more causative factors mentioned above. The condition is present in about 1% of the general male population [5]. However, its prevalence is much higher among infertile men, impacting up to 15% of individuals and significantly reducing a couple’s chance of conception [5]. The initial diagnosis of azoospermia should be corroborated by at least one additional semen analysis following centrifugation. The minimum time between collections has not been defined. This step is essential, as sperm may be discovered in the pellet of up to 35% of men who were initially diagnosed with azoospermia [6].

Once the laboratory diagnosis of azoospermia is established, focus should be placed on identifying possible etiologies through a comprehensive history and physical examination, laboratory tests, imaging, and genetic studies [7]. On this basis, azoospermia can be classified into two broad categories: obstructive azoospermia and nonobstructive azoospermia (NOA). In obstructive azoospermia (post-testicular azoospermia), the spermatogenesis is normal, and the obstruction results from bilateral obstruction of the seminal ducts [8]. By contrast, NOA is associated with pre-testicular or testicular disorders that result in spermatogenic failure [9]. In a cohort of 8568 men seeking fertility and attending our tertiary center for male reproductive health, 1003 (11.7%) had a NOA diagnosis [10].

**Figure 1:**
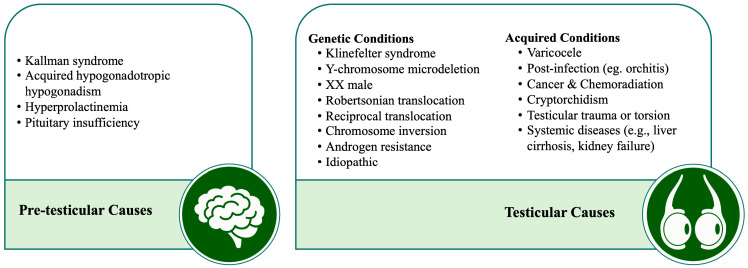
Etiology of non-obstructive azoospermia.

Several causes for NOA have been identified and are classified as pre-testicular or testicular (Figure 1) [11,12]. Pre-testicular causes are mainly endocrine-related, resulting from disruptions in the hypothalamic–pituitary–gonadal axis, leading to secondary testicular dysfunction. These cases can be genetic or non-genetic and include conditions like hypogonadotropic hypogonadism (congenital or acquired), hyperprolactinemia, and androgen resistance. Testicular causes can also be genetic, including Klinefelter syndrome (KS), Y chromosome microdeletion (YCMD), chromosomal translocations or inversions, or non-genetic (acquired) conditions such as varicoceles, testicular infections or inflammatory conditions, malignancies or post-chemoradiation effects, cryptorchidism, and testicular trauma. Despite thorough investigations, no clear cause is identified in many cases, which are then classified as idiopathic [12,13]. In a cohort of 767 patients with testicular NOA attending our center, idiopathic was found to be the prevailing etiology (69.4%), followed by cryptorchidism (15.0%), genetic defects (5.6%), postgonadotoxic therapy (5.1%), postinfection (4.4%), and post-trauma (0.5%) [10].

Historically, patients with testicular NOA were deemed sterile, and adoption was their only option for parenthood. This condition could significantly impact a couple’s psychological well-being, often leading to anxiety and depression. These challenges were further intensified by the invasive nature of treatments and the uncertainty of their outcomes. However, the advent of intracytoplasmic sperm injection (ICSI) and testicular sperm retrieval in the 1990s have allowed NOA patients to father biological children [14,15]. These patients must receive thorough evaluations by specialists in male reproduction for many reasons [16]. First, potentially correctable conditions causing or contributing to NOA, such as endocrine disorders, varicoceles, and exposure to toxicants, can be identified. Second, conditions that are irreversible but suitable for ICSI using the patient’s own sperm can be determined. Third, situations such as XX karyotype and complete microdeletions of azoospermia factor (AZFa microdeletion and/or AZFb microdeletion on the Y chromosome), for which donor insemination or adoption are the only solutions, might be found.

Moreover, a complete andrological evaluation might help identify health-threatening conditions or coexistent diseases potentially contributing to fertility impairment in NOA males that require medical care, such as obesity, metabolic syndrome, erectile dysfunction, hypogonadism, kidney diseases, and cancer. Lastly, a well-conducted evaluation can reveal conditions that might affect patient or offspring health (Klinefelter syndrome, AZFc microdeletion), and the affected couples should be counseled accordingly. Equally important is the role of the reproductive urologist/andrologist in recommending and performing the most optical sperm retrieval (SR) procedure to enhance the likelihood of retrieving testicular sperm. In this regard, microsurgical expertise during testicular sperm retrieval is among the prerequisites for a successful surgical outcome.

This review aims to summarize the available evidence concerning the efficacy of ICSI in NOA males with testicular causes (from now on termed NOA), describe the predictors of successful ICSI outcomes in this patient population, and present the evidence concerning the consequences of ICSI for the health of the offspring of NOA fathers. We also discuss potential interventions to reduce health risks to offspring health.

## 2. Intracytoplasmic Sperm Injection (ICSI) Explained

The fertilization of oocytes with male gametes in vitro was a groundbreaking advancement that helped overcome many forms of female infertility [17]. However, shortly after its implementation, the limitations of in vitro fertilization (IVF) became apparent, particularly for couples with poor semen quality [18]. Various techniques were employed to improve fertilization rates with IVF, including sperm selection strategies (such as multilayer density gradients and swim-up techniques) [19], sperm motility enhancement [20], and modifications of the zona pellucida [21,22]. However, it was not until the introduction of ICSI that assisted reproduction expanded to include male patients with severe spermatogenic dysfunction [23].

**Figure 2:**
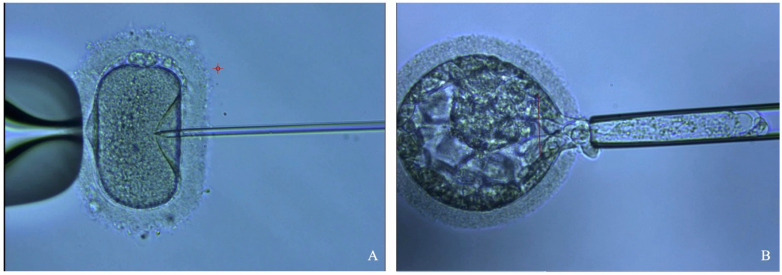
(**A**) Intracytoplasmic sperm injection; (**B**) trophectoderm biopsy for preimplantation genetic testing.

Unlike conventional IVF, which generally depends on the sperm’s natural fertilizing ability, during ICSI, a single spermatozoon is meticulously selected and injected into the oocyte’s cytoplasm using a micropipette (Figure 2). The injected oocyte is then monitored, and the resulting embryo is either transferred to the uterine cavity ~3–5 days after injection or cryopreserved.

Given that ICSI can be performed using sperm of suboptimal quality, its application has been extended to include testicular sperm obtained from men with NOA [23]. This was first demonstrated by Devroey et al. [24] in 15 NOA men. Testicular sperm retrieval was performed using an open biopsy on the same day as oocyte retrieval. The extracted specimens were examined, and an additional sample was sent for histological evaluation. In 13 out of 15 patients, small numbers of spermatozoa were found, and all specimens exhibited severe spermatogenic defects, confirmed by histopathology. In this series, testicular sperm injections resulted in a 47.8% fertilization rate. A total of 32 embryos were transferred, resulting in three pregnancies—one set of triplets, one set of twins, and one singleton—with an overall implantation rate of 18.7% [24].

Subsequent studies compared ICSI outcomes between patients with NOA, obstructive azoospermia (OA), and non-azoospermic infertile males. In one study by the authors’ group [25], 370 azoospermic and 465 non-azoospermic patients were included. The authors found significantly lower fertilization (43.7%), clinical pregnancy (28.6%), and live birth rates (21.4%) in men with NOA compared to those with OA (62.9%, 48.9%, 37.5%) and in ejaculated sperm (64.5%, 41.7%, 32.3%). In this study, the authors also conducted a systematic review of 20 publications comparing reproductive outcomes of males with NOA and OA, with or without a control group of non-azoospermic males, revealing marked inconsistencies in the reported outcomes. Some studies reported decreased pregnancy rates (clinical or live births), while others showed similar outcomes among the studied groups. Despite these conflicting results, this review demonstrated that ICSI can be applied to men with NOA and confirmed that pregnancy and live birth, though with lower success rates, are achievable with ICSI using testicular sperm.

## 3. Realistic Expectations in ICSI for Non-Obstructive Azoospermia

### 3.1. Factors Influencing Successful Sperm Retrieval in NOA Patients

Several factors can influence the reproductive outcome of patients with NOA undergoing ICSI. Before exploring clinical considerations, it is crucial to advocate for and ensure the adoption of healthy lifestyle habits by NOA patients before they receive medical treatment. These habits include weight reduction, regular physical exercise, and consumption of nutrient and antioxidant-rich diets, as these measures can help reduce oxidative stress, which is harmful for the testicular microenvironment. Successful testicular sperm retrieval is crucial for ICSI, making it essential to understand the predictors of successful sperm retrieval.

#### 3.1.1. Sperm Retrieval Technique

**Figure 3:**
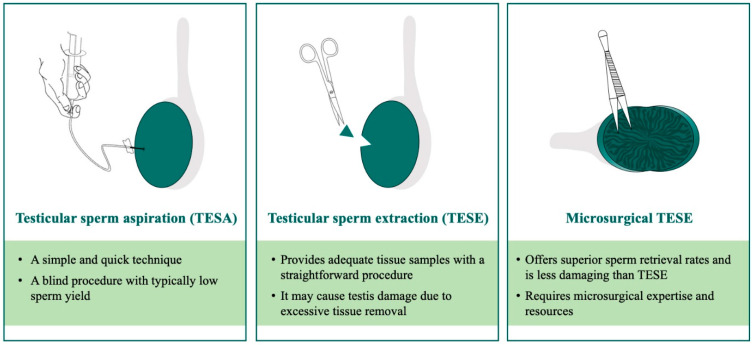
Testicular sperm retrieval techniques.

Studies using testicular biopsy results have identified a mixed histopathologic pattern in men with NOA, where various tubular histologies are seen, including minute foci of normal spermatogenesis in some cases [24,26]. This finding led to the development of several sperm retrieval procedures, such as testicular sperm aspiration (TESA), conventional testicular sperm extraction (TESE), and microsurgical TESE (Figure 3). TESA is a percutaneous fine-needle aspiration and biopsy procedure to retrieve fluid and testicular tissue [27,28]. While it is a simple and quick procedure, TESA might miss pockets of spermatogenesis due to its random sampling nature. Conventional TESE involves incision of the tunica albuginea and excision of the protruding testicular parenchyma [24,29]. Although commonly used, conventional TESE poses a risk of disproportionate tissue damage or loss due to devascularization or excessive excision of testicular tissue [30]. On the other hand, microsurgical TESE offers a magnified dissection of testicular tissue and meticulous sampling of dilated tubules, which are more likely to harbor sperm [31]. As such, microsurgical TESE is a more precise sperm retrieval technique that is relatively less damaging to testicular tissue and its function [32,33].

Several studies compared sperm retrieval rates among the above-mentioned methods, which were appraised by multiple systemic reviews [34,35,36]. Overall, the highest sperm retrieval rate has been observed with microsurgical TESE (40–60%) compared to conventional TESE (30–40%) and TESA (20–30%) [37,38]. The superior sperm retrieval outcome, along with its testicular parenchyma-sparing nature, makes microsurgical TESE the gold standard approach for testicular sperm retrieval in NOA patients. However, the procedure is technically demanding and requires master surgical expertise, which may limit its generalizability, thus underscoring the importance of specialized treatment for this patient population.

#### 3.1.2. Other Clinical and Laboratory Factors

Since the introduction of testicular sperm retrieval procedures, numerous studies explored the role of clinical and laboratory variables in predicting the likelihood of a successful outcome. Although a detailed discussion of these factors is beyond the scope of this paper, factors such as patient age, testicular volume, serum FSH, inhibin and testosterone levels, genetic background, surgical history, and testicular histopathology pattern have been the most investigated [37].

Among clinical factors, Klinefelter syndrome (KS) seems to be a negative predictor for sperm retrieval success, whereas larger testicular volume is associated with increased retrieval rates [37]. Laboratory tests offer limited clinical value, except for Y chromosome microdeletion (YCMD) screening. Patients with deletions involving the azoospermia factor (AZF) a region and/or AZFb region should be discouraged from pursuing sperm retrieval, as success rates are virtually non-existent [37]. In comparison, patients with AZFc deletions have a success rate reaching up to 62% [39,40]. Among all factors, testicular histopathology appears to be highly influential. The highest retrieval rates are observed in men with hypospermatogenesis (HS) (50–100%), followed by maturation arrest (MA) (10.8–77.3%) [37]. The presence of Sertoli cell-only (SCO) syndrome, on the other hand, confers a poor prognosis, with sperm retrieval rates ranging between 29.1 and 60% [37].

In a recent study, our group demonstrated that SR success by micro-TESE in NOA patients is negatively associated with biochemical hypogonadism, characterized by low circulating total testosterone levels [10]. In another recent report, we found that among hypogonadal NOA males, baseline FSH levels, pre-SR hormonal stimulation, clinical varicocele, a history of previous varicocelectomy, and testicular histopathology were independent predictors of SR success [41]. In particular, lower baseline FSH levels and a history of prior varicocelectomy were associated with increased odds of successful SR, while a clinical varicocele decreased these odds. Patients exhibiting biopsies indicative of hypospermatogenesis or MA had significantly higher odds of successful SR than those with SCO. Additionally, hormone-pretreated hypogonadal NOA patients achieved higher SR success rates than their hormone-untreated counterparts. Interestingly, this study showed that in hypogonadal NOA men, hormonal stimulation with exogenous gonadotropins and the absence of a clinical varicocele are associated with improved micro-TESE success, offering potential treatment insights. These findings suggest that pre-sperm retrieval interventions might improve SR outcomes, thus opening the possibility to explore such strategies in well-characterized patient subgroups [41,42,43].

### 3.2. Factors Influencing Pregnancy Outcome with ICSI

Following successful sperm retrieval, several factors may influence the pregnancy outcome of ICSI in patients with NOA.

#### 3.2.1. Sperm Quality and Quantity

The quality and quantity of sperm retrieved from patients with NOA are pivotal for the outcome of ICSI. In theory, obtaining a higher number of sperm during sperm retrieval should provide more choices for sperm selection, ultimately resulting in a higher number of successful microinjections. However, several factors, including the surgical technique, testicular histopathology, and previous surgical interventions, might influence the retrieved sperm quality and quantity.

While a higher sperm retrieval rate is generally achieved with microsurgical TESE than conventional TESE or TESA, it is challenging to determine which method offers the highest yield, as most published studies in the literature consider finding a single spermatozoon a positive sperm retrieval outcome. Nonetheless, microsurgical TESE is superior to other methods, particularly in patients with severe testicular histopathology phenotypes such as Sertoli cell-only syndrome [44].

Sperm quality is mainly influenced by the handling maneuvers employed following retrieval (see next section). The primary objective is to inject viable, motile, and morphologically normal sperm. However, this is not always achievable, as in many instances, only non-motile or morphologically abnormal sperm are retrieved. Yet, pregnancies following ICSI using testicular non-motile sperm from azoospermic patients have been reported. In one report, Shulman et al. [45] compared the results of 19 ICSI cycles performed with non-motile testicular sperm from azoospermic men to 34 cycles using motile testicular sperm. While a significantly lower fertilization rate was observed in patients with immotile testicular sperm (51%) compared to those with motile testicular sperm (62%), the pregnancy rates were similar in both groups (15.8% vs. 23.5%).

#### 3.2.2. Laboratory Techniques

Preparing testicular tissue from NOA patients for assisted reproductive technology (ART) involves a meticulous search to identify and select viable sperm for fertilization. The obtained testicular tissues are initially processed mechanically using techniques such as shredding and mincing to release sperm from the seminiferous tubules into a medium [46]. These methods allow for direct examination of the obtained suspension under high magnification. In cases where the sperm yield is very low or absent, enzymatic digestion using collagenase type IA or type IV can break down the extracellular matrix and basement membrane of the testicular tissue, potentially improving sperm recovery [47,48]. The excised testicular tissue is often contaminated with red blood cells, making visualization of immotile sperm challenging. An erythrocyte lysing buffer can be added to the obtained suspension after mincing, improving sperm identification without affecting the sperm fertilizing potential [49].

Some authors have reported pregnancies following intracytoplasmic injection of elongated or round spermatids in cases of negative sperm retrieval outcomes. Elongated spermatids are easily identified, unlike round spermatids. Intracytoplasmic injection of elongated or round spermatids results in far fewer favorable outcomes than the injection of mature sperm [50]. Furthermore, injecting spermatids raises safety concerns due to the potential genetic or epigenetic risks to the offspring related to unstable or damaged spermatid DNA contents [50].

Other laboratory procedures can assist embryologists in selecting viable immotile sperm for injection [51]. These include culturing the obtained tissue suspension in media containing motility enhancers such as phosphodiesterase inhibitors, pentoxifylline, or theophylline [52]; the use of a hypoosmotic swelling test [53]; sperm tail flexibility test [54]; intracytoplasmic morphologically selected sperm injection (IMSI) [55]; laser-assisted sperm selection [56]; birefringence-based sperm selection [57]; and microfluidics-assisted sperm sorting [58].

Motility enhancers prevent the degradation of cyclic adenosine monophosphate (cAMP), the primary signal for the onset of progressive sperm motility [59]. The hypoosmotic swelling test assesses the functional integrity of the sperm membrane, which swells upon incubation in a hypo-osmotic solution [53]. Sperm tail flexibility observes sperm tail movement by mechanical agitation with a lateral touch of the microinjection pipette [54]. IMSI utilizes ultra-high magnification to select sperm with normal nuclear morphology [55], while birefringence-based selection assesses the structural integrity of the sperm nucleus and acrosome complex, allowing for the selection of mature sperm with a characteristic intrinsic birefringent appearance [57]. Laser-assisted sperm selection involves targeting the tip of the sperm’s tail with a quick, 2-millisecond burst using 200 µJ of energy [56]. The resulting tail curl confirms the sperm’s viability, qualifying them for use in ICSI procedures. A newly developed microfluidic system was introduced to extract sperm from testicular samples [58]. The system employs two sequential modules to process the testicular tissue extract; the initial module uses a spiral microchannel to apply inertial forces to segregate sperm from red blood cells and other cellular particles, and the subsequent hollow fiber membrane module isolates other cells and extracts excess media, thereby enhancing the sperm concentration in the suspension.

#### 3.2.3. Fresh vs. Frozen-Thawed Sperm

Research on the outcomes of ICSI using fresh and frozen-thawed testicular sperm in men with NOA has been a significant focus within reproductive technology. Importantly, multiple studies suggest no significant differences in fertilization rates, clinical pregnancy rates, and live birth rates between the use of fresh or frozen-thawed sperm [60,61,62,63].

Testicular sperm can be preserved as whole biopsies or shredded tissue suspensions and, more recently, vitrified individually or in small groups [64,65]. Crabbe et al. [66] have shown that freezing sperm as a suspension is more effective in preserving sperm motility and vitality than freezing whole biopsies. Laursen et al. demonstrated that pregnancy could be achieved from a single testicular spermatozoon frozen by vitrification on Cell-Sleeper devices [65]. Nogueira et al. [67], studying the structural changes in sperm due to the freezing and thawing process, observed swelling and rupture in sperm membranes under microscopic examination, yet these alterations did not impact fertilization and pregnancy rates.

These promising insights indicate that sperm freezing offers significant logistical benefits and can decrease the risks linked to timing sperm retrieval with oocyte retrieval without diminishing the effectiveness of ICSI procedures.

#### 3.2.4. Female Partner Health

The health of the female partner significantly affects reproductive outcomes in couples undergoing ICSI, especially if the male partner has NOA. Studies have underscored factors such as the female partner’s age and ovarian reserve as crucial to determine the success of ICSI in this patient population [68].

Maternal age has long been accepted as a critical determinant of ICSI outcomes. Research consistently shows an inverse relationship between oocyte retrieval, fertilization, embryo quality, pregnancy rates, and the age of women undergoing ICSI [69,70]. In cases where the male partner has NOA, these factors become even more pivotal because the sperm used is already compromised in terms of its ability to fertilize the oocyte [71].

Freidler et al. [72] explored predictors of a successful ICSI outcome from 192 cycles in patients with azoospermia (OA or NOA). The authors observed that the likelihood of pregnancy was significantly reduced for female partners aged over 38 years and/or those with poor ovarian reserve when the number of mature oocytes available for injection was four or fewer. Further evidence is provided by analyzing the large dataset from the Assisted Reproductive Technology Clinic Outcome Reporting System (SART CORS) registry [73]. In this study, 24,763 ICSI cycles using fresh autologous oocytes and surgically retrieved sperm were examined. The outcomes were compared between women aged <30, 30–34, 35–38, 38–42, and >42 years, revealing that older women underwent significantly longer stimulation periods and had fewer oocytes retrieved, as well as two-pronuclei (2PN) zygotes. Both the clinical pregnancy rates and live birth rates declined with increasing maternal age, with live birth rates ranging from 50.4% in women under 30 years to 7.2% in women aged over 42 years.

Previous studies also found that female age was the most relevant factor in predicting the probability of a blastocyst being euploid [67]. However, the prediction was negatively modulated if testicular sperm from men with NOA were used [67]. These findings indicate that an increased number of mature oocytes is needed to counteract the effect of microinjecting testicular sperm from men with NOA [67]. On this basis, a calculator was created [67] and validated [74,75] to estimate the minimum number of metaphase II oocytes needed to obtain at least one euploid blastocyst for transfer in couples undergoing IVF/ICSI (freely available at https://art-one.merckgroup.com/art, accessed on 15 July 2024).

## 4. Offspring Health

**Figure 4:**
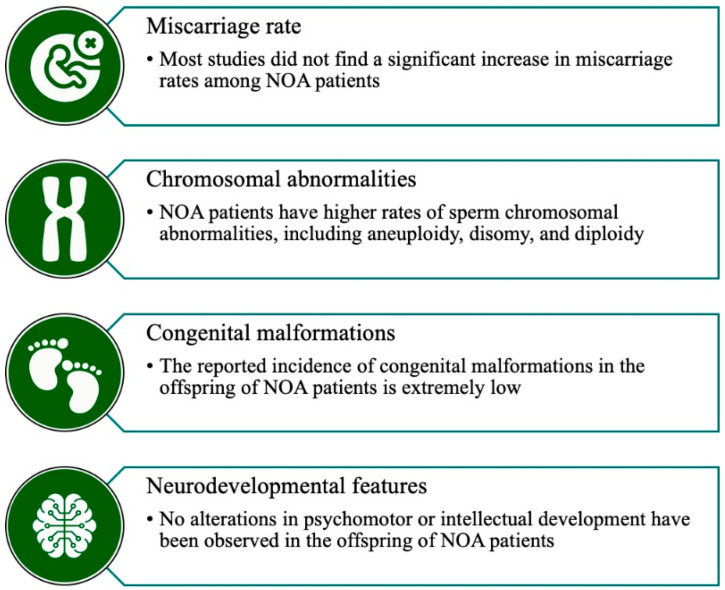
Outcomes of intracytoplasmic sperm injection on the health of offspring from patients with non-obstructive azoospermia.

The use of ICSI for men with NOA has raised concerns about the health of the resulting offspring owing to uncertainties related to the integrity of the genome and epigenome of testicular sperm [23]. Numerous studies have included offspring health data following ICSI in males with NOA, summarized below (Figure 4).

### 4.1. Miscarriage

A total of 36 original studies were identified that reported miscarriages following ICSI in men with NOA (Table 1). Most of these studies were retrospective, except for two prospective studies [76,77]. Seventeen studies included men with NOA without a comparator group, and overall, their results indicate a low risk of miscarriage in this patient population [76,78,79,80,81,82,83,84,85,86,87,88,89,90,91,92]. Several of these studies examined specific factors that could influence miscarriage rates, such as the use of fresh vs. frozen-thawed sperm, motile vs. immotile sperm, the underlying etiology of NOA, histopathology results, sperm retrieval methods, and whether a concurrent varicocele was treated before ICSI.

Five studies explored the impact of using cryopreserved testicular sperm on miscarriage rates, with four studies reporting no statistically significant difference [80,81,82,83,84]. Only Zhang et al. (2021) observed a significantly higher miscarriage rate using frozen-thawed sperm (23.8%) compared to fresh sperm (0%; (*p* = 0.009)) [80]. Three studies assessed the effect of sperm motility with or without cryopreservation, revealing no significant differences between motile and immotile sperm, whether fresh or frozen-thawed [85,86,93]. Additionally, Giorgetti et al. found no significant differences in miscarriage rates among patients with different testicular histopathologies [76].

Two studies explored the miscarriage rate following different sperm retrieval methods, TESA vs. TESE [89] or TESE vs. microsurgical TESE [90], and reported no significant differences between the approaches. Inci et al. retrospectively compared the outcomes of 66 NOA patients who had varicocele ligation vs. 21 patients without varicocele ligation before ICSI, finding no difference in miscarriage rates between the two groups [91]. Zhang et al. [81] compared outcomes among patients with KS, AZFc, cryptorchidism, mumps orchitis, and idiopathic NOA, reporting no differences in miscarriage rates among groups.

Nineteen studies compared ICSI miscarriage rates between NOA patients and a comparator group that constituted patients with OA [72,75,77,94,95,96,97,98,99,100,101,102,103,104,105,106,107,108,109]. Six studies included additional comparative groups of men with varying spermatogenic dysfunctions or normal semen parameters [75,77,106,107,108,109].

Most of these studies found no significant differences in miscarriage rates between groups. Only two studies reported significantly higher miscarriage rates in men with NOA compared to men with OA [97,98]. In a cohort study by our group [106], including 151 NOA patients, 146 OA patients, and 40 fertile donors, we reported 48 infants delivered after ICSI with testicular sperm from NOA patients. Of these, 18 deliveries were singletons (58.1%), 9 were twins (29%), and 4 were triplets (12.9%). The miscarriage rates did not differ among NOA (28.6%, 12/42), OA (23.9%, 16/67) patients, and users of donor sperm (25%, 5/20) (*p* = 0.88). Furthermore, fresh or frozen-thawed testicular sperm for ICSI yielded similar miscarriage rates between NOA and OA patients [72,94,95,96]. Even studies including men with normal sperm parameters did not detect any significant differences in miscarriage rates following ICSI with testicular sperm from NOA men and with ejaculated sperm [75,106,107].

**Table 1:** Studies evaluating miscarriage rates in couples undergoing ICSI with testicular sperm of patients with non-obstructive azoospermia.

Reference #	Study	Design	Study Group (n)	Control Group (n)	Outcome Measures	Findings	
						NOA-ICSI	NOA-ICSI vs. Comparator
[83]	Friedler et al., 1997	Retrospective	Testicular sperm from NOA patients (18) by TESE Fresh sperm (9) Cryopreserved sperm (9)	NR	Miscarriage rate	Fresh sperm: Two twin pregnancies and four singleton pregnancies. One of the twin pregnancies resulted in a spontaneous miscarriage during the first trimester (1/6). Cryopreserved sperm: One twin pregnancy and one singleton pregnancy out of three ended in a spontaneous miscarriage during the first trimester (2/3). There was no statistically significant difference in miscarriage rates between the use of fresh and cryopreserved sperm.	NR
[84]	Ben-Yosef et al., 1999	Retrospective	Testicular sperm from NOA patients (25) by TESE Fresh sperm (14) Cryopreserved sperm (11)	NR	Miscarriage rate	Of the nine pregnancies achieved (four from fresh and five from cryopreserved spermatozoa), two were missed abortions (group not specified), one was ectopic, and six resulted in deliveries of healthy babies (three of each group).	NR
[94]	Habermann et al., 2000	Retrospective	TESE-ICSI cycles from NOA patients (12) Fresh sperm (3) Frozen-thawed sperm (9)	TESE-ICSI cycles from OA patients (34): Fresh sperm (9); Frozen-thawed sperm (25).	Miscarriage rate	TESE-ICSI cycles with fresh sperm of NOA patients: There was one pregnancy and one delivery of singletons. TESE-ICSI cycles with frozen-thawed sperm of NOA patients: There were six pregnancies and two miscarriages.	The miscarriage rates were similar between OA and NOA patients for fresh (25%, 1/4) and frozen-thawed sperm (30.8%, 4/9).
[89]	Mercan et al., 2000	Retrospective	Testicular sperm from NOA patients (291) By TESA (63) By TESE (228)	NR	Miscarriage rate	The miscarriage rates according to testicular sperm retrieval method were: TESA: 20.7% (6/29); TESE: 24.2% (16/66) (*p* = NS).	NR
[72]	Friedler et al., 2002	Retrospective	Testicular sperm from NOA patients (123) by TESE Fresh sperm (65 ICSI cycles) Frozen-thawed sperm (63 ICSI cycles)	Epidydimal sperm from OA patients (52) By PESA Fresh sperm (55 ICSI cycles) Frozen-thawed sperm (80 ICSI cycles)	ICSI outcomes	There was no significant difference between early miscarriage rate in NOA patients using testicular fresh sperm (15.7%, 3/19) compared to frozen-thawed testicular sperm (21%, 4/19).	The pregnancy rate/embryo transfer, early miscarriage rate, and ongoing/delivery pregnancy rates were similar in both groups using fresh or frozen-thawed sperm for ICSI for OA and NOA patients.
[97]	Pasqualotto et al., 2002	Retrospective	ICSI infants from NOA patients (68 cycles) by TESA	ICSI infants from OA patients (130 cycles) By PESA	Miscarriage rate	The miscarriage rate was higher in those in whom immotile vs. motile spermatozoa were retrieved (70% vs. 25.5%, *p* < 0.05).	NOA patients showed higher miscarriage rates (40%) than OA patients (28%) (*p* = 0.01).
[98]	Pasqualotto et al., 2005	Retrospective	TESA-ICSI cycles from NOA patients (102)	PESA-ICSI cycles from AO patients (155): Post-vasectomy (99); Congenital (25); Post-infection (31).	ICSI outcomes	From 102 TESA-ICSI cycles of NOA patients, 22 pregnancies were achieved, and 10 miscarriages occurred (45.6%).	No statistical difference was noted among groups despite NOA patients showing lower pregnancy rates. However, miscarriage rates were higher in NOA patients (45.6%) compared with other groups: Post-vasectomy (25.8%) Congenital (28.6%) Post-infection (28.6%) (*p* = NS)
[76]	Giorgetti et al., 2005	Prospective	TESE-ICSI cycles performed in NOA patients (99) SCO (16) MA (35) Hypo (48)	NR	Miscarriage rate	There was no significant difference between testicular histopathology groups in miscarriage rates: SCO: 1/5; MA: 3/13; Hypo: 2/17. From 35 pregnancies achieved with fresh embryos: Five spontaneous first-trimester miscarriage and one second-trimester miscarriage. From two pregnancies achieved with frozen-thawed embryos: No miscarriages were reported.	NR
[95]	Wu et al., 2005	Retrospective	TESE-ICSI cycles performed in NOA patients (30) Fresh sperm (6) Frozen-thawed sperm (24)	TESE-ICSI cycles performed in OA patients (28) Fresh sperm (16) Frozen-thawed sperm (12)	Miscarriage rate	From six TESE-ICSI cycles with fresh sperm of NOA patients, there were two clinical pregnancies and no miscarriages reported (0%, 0/2). From 24 TESE-ICSI cycles with frozen-thawed sperm of NOA patients, there were 15 clinical pregnancies, and 5 miscarriages reported (33.3%, 5/15).	Both fresh and frozen-thawed TESE-ICSI cycles had similar spontaneous miscarriage rates (25% vs. 19.5%, *p* = NS) for OA and NOA patients.
[85]	Konc et al., 2006	Retrospective	TESE-ICSI cycles performed in OA and NOA patients (167) by TESE Fresh sperm (68) Frozen sperm (99) Motile sperm (50) Immotile sperm (117)	NR	Miscarriage rate	No difference was found in the abortion rates: Fresh sperm (10/20, 50%); Frozen sperm (7/22, 32%); Motile sperm (6/14, 43%); Immotile sperm (11/28, 39%) (*p* = NS).	NR
[86]	Konc et al., 2008	Retrospective	TESE-ICSI cycles from NOA patients Fresh/Motile sperm (30) Fresh/Immotile sperm (34) Frozen/Motile sperm (19) Frozen/Immotile sperm (74)	NR	Miscarriage rate	No differences were found in the abortion rates: Fresh/motile (4/10, 40%); Fresh/immotile (6/10, 60%); Frozen/motile (2/4, 50%); Frozen/immotile (6/18, 33%) (*p* = NS)	NR
[90]	Ravizzini et al., 2008	Retrospective	NOA patients (53)	NR	Miscarriage rate	From 32 patients with positive micro-TESE, 13 couples achieved clinical pregnancy, and none of them suffered a miscarriage.	NR
[91]	Inci et al., 2009	Retrospective	Micro-TESE-ICSI infants from NOA patients (87) Varicocele treated (66) Varicocele untreated (21)	NR	Miscarriage rate	The miscarriage rates did not differ significantly between treated varicocele (18.2%, 2/11) and untreated varicocele patients (0%, 0/2) (*p* = NS).	NR
[99]	Semião-Francisco et al., 2010	Retrospective	NOA patients (102) by TESA	OA patients: TESA (103) PESA (171)	Miscarriage rate	The miscarriage rates did not differ significantly between OA-TESA and NOA-TESA patients (*p* = NS).	The miscarriage rates were significantly higher for patients with OA who underwent TESA as compared to PESA (*p* = 0.038).
[96]	Kalsi et al., 2010	Retrospective	Testicular sperm from NOA patients (48) Fresh sperm (41) Frozen-thawed sperm (7)	Epidydimal sperm from OA patients (215): Fresh sperm (173) Frozen-thawed sperm (42) Testicular sperm from OA patients (43): Fresh sperm (28) Frozen-thawed sperm (15)	ICSI outcomes	In NOA patients, there was a difference between frozen-thawed sperm and fresh sperm concerning pregnancy rates, live birth rate, and miscarriage rate (*p* = NS). While with fresh sperm the miscarriage rate was 13.3% (2/15), using frozen-thawed sperm there was no miscarriage reported (0%, 0/4) (*p* = NS).	When comparing groups, there were no significant differences in fertilization, pregnancy, and live birth rates.
[100]	He et al., 2010	Retrospective	ICSI cycles performed in NOA patients (42)	ICSI cycles performed in OA patients (112)	ICSI outcomes	From 42 ICSI cycles of NOA patients, nine pregnancies were achieved (21.4%), and three miscarriages occurred (33.3%).	Although the clinical pregnancy rate was higher in OA patients compared with NOA patients (40.2% vs. 21.4%, *p* < 0.05), the miscarriage rates did not differ among the groups (15.6% vs. 33.3%, *p* = NS).
[77]	Tehraninejad et al., 2011	Prospective	NOA patients (134) Testicular sperm by micro-TESE	Oligozoospermic patients (314) Ejaculated sperm OA patients (180) Epidydimal sperm by PESA	Miscarriage rate	From 134 micro-TESE-ICSI cycles of NOA patients, the fertilization rate was 51.8%, the clinical pregnancy rate was 13.4%, and the miscarriage rate was 8%.	The frequency of miscarriage from men with NOA (8%) was similar compared to oligozoospermic (10.7) and OA (9.7%) patients (*p* = NS).
[88]	Cavallini et al., 2011	Retrospective	TESE-ICSI cycles performed in NOA patients (184)	NR	Miscarriage rate	From 184 ICSI cycles, 14 pregnancies were achieved, and 1 miscarriage occurred.	NR
[87]	Boitrelle et al., 2011	Retrospective	TESE-ICSI cycles performed in NOA patients (280)	NR	Miscarriage rate	Of the 38 pregnancies, three suffered a miscarriage before the first trimester of pregnancy.	NR
[101]	Abdel Raheem et al., 2013	Retrospective	TESE-ICSI cycles from NOA patients (77) Hypo (27) MA (20) SCO (18)	TESE-ICSI cycles from OA patients (60)	ICSI outcomes	There were no statistically significant differences in any of the ICSI outcomes measures (fertilization rate, embryo cleavage rate, clinical pregnancy rate, live birth rate, miscarriage rate) between different testicular histopathologies of NOA patients.	There were no statistically significant differences in any ICSI outcomes when using fresh and frozen-thawed sperm from OA or NOA patients. Additionally, ICSI outcomes did not differ between cycles that used or did not use pentoxifylline for motility enhancement.
[102]	Celikten et al., 2013	Retrospective	TESE-ICSI cycles of NOA patients (133)	PESA-ICSI cycles of OA patients (78)	ICSI outcomes	From 133 TESE-ICSI of NOA patients, 26 pregnancies were achieved (19.5%), and 16 miscarriages occurred (61.5%).	There were no significant differences in clinical pregnancy (16/78 vs. 26/133, *p* = NS) and miscarriage rates (10/16 vs. 16/26, *p* = NS) in OA and NOA patients.
[103]	Karacan et al., 2013	Retrospective	Testicular sperm from NOA patients (209) by micro-TESE Only motile spermatozoa	Testicular sperm from OA patients (128) by TESE Only motile spermatozoa	ICSI outcomes	The miscarriage rates for NOA patients were similar whether using fresh sperm (6.8%, 2/23), sperm used 24 h later (12.5%, 1/8), or frozen-thawed sperm (10%, 2/20), with no statistically significant differences (*p* = NS).	There were no statistically significant differences in any parameters (implantation rate and miscarriage) among the groups (*p* = NS).
[82]	Madureira et al., 2014	Retrospective	Testicular sperm from NOA patients with KS (65) by TESE: Fresh sperm (19) Frozen-thawed sperm (13)	NR	Miscarriage rate	The miscarriage rates of NOA patients with KS did not differ from using fresh sperm (16.7%, 2/12) compared with frozen-thawed sperm (0%, 0/4) (*p* = NS).	NR
[92]	Karacan et al., 2014	Retrospective	NOA patients (86) TESE (47) Micro-TESE (39)	NR	Miscarriage rate	From 12 clinical pregnancies, there was only 1 case of miscarriage using TESE sperm retrieval. The only pregnancy achieved by micro-TESE ended in full-term delivery.	NR
[106]	Esteves et al., 2014	Retrospective	Testicular sperm from NOA patients (151) by micro-TESE	Testicular sperm from OA patients (146) by TESA Ejaculated sperm from donors (40)	ICSI outcomes	From 48 infants delivered after ICSI with testicular sperm from NOA patients, 18 deliveries were singletons (58.1%), 9 were twins (29%), and 4 were triplets (12.9%).	The miscarriage rates did not differ among NOA (28.6%, 12/42), OA (23.9%, 16/67) patients and donor sperm (25%, 5/20) (*p* = NS).
[93]	Hessel et al., 2015	Retrospective	TESE-ICSI cycles (745) from NOA patients (61%) and OA patients (39%) Motile sperm (586) Immotile sperm—tail touch (159)	NR	Miscarriage rate	There was no significant difference in abortion rates between motile spermatozoa (24%) compared with tail touch spermatozoa (38%, *p* = 0.08).	NR
[75]	Mazzilli et al., 2017	Retrospective	TESE-ICSI of NOA patients (49)	TESA-ICSI of OA patients (34) ICSI infants with ejaculated sperm from: OAT (188); Moderate male factor (420); Normozoospermic (528).	ICSI outcomes	From 49 TESE-ICSI cycles of NOA patients, 7 pregnancies were achieved and 1 case of miscarriage (14.3%).	There were no statistically significant differences among groups in biochemical pregnancy and miscarriage rates.
[104]	Bocca et al., 2017	Retrospective	NOA patients (8)	OA patients (44)	Miscarriage rate	Miscarriage rates between OA and NOA groups were not significantly different (10.7% vs. 23.1%, *p* = NS). Maternal age <35 or >35 had no significant impact on these results (*p* = NS).	NR
[109]	Okuyama et al., 2017	Retrospective	NOA patients (388), including AZFc (28) and KS (83)	Cryptozoospermia (58) OA (272)	Miscarriage rate	The frequency of miscarriage from men with NOA was similar comparing fresh oocytes and fresh sperm/fresh oocytes and frozen-thawed sperm/frozen-thawed oocytes and fresh sperm (*p* = NS).	The frequency of miscarriage was similar comparing all groups (*p* = NS).
[79]	Zhang et al., 2021	Retrospective	NOA patients (65) who underwent 70 ICSI cycles 40 ICSI cycles with fresh spermatozoa (group A); 30 ICSI cycles with cryopreserved spermatozoa (group B).	NR	Miscarriage rate	There were significantly higher miscarriage rates in group B with cryopreserved spermatozoa (23.8%) than in group A with fresh spermatozoa (0%) (*p* = 0.009).	NR
[105]	Vahidi et al., 2021	Retrospective cross-sectional study	Testicular sperm from NOA patients (138)	Testicular sperm from OA patients (172)	Miscarriage rate		There was no difference in miscarriage rates between OA (7/172, 4.0%) and NOA (5/138, 3.6%) patients (*p* = NS).
[81]	Zhang et al., 2021	Retrospective	Micro-TESE-ICSI cycles performed in NOA patients (347) KS (125) AZFc (64) Cryptorchidism (39) Mumps and orchitis (23) Idiopathic (96)	NR	Miscarriage rate	No differences were found in the miscarriage rates among all groups (*p* = NS).	NR
[80]	Zhang et al., 2021	Retrospective	Micro-TESE-ICSI cycles performed in NOA patients (344) Fresh sperm (234) Frozen-thawed sperm (110)	NR	Miscarriage rate	The miscarriage rate using fresh sperm was 6.0% (7/116) while the rate using frozen-thawed sperm was 14.9% (7/47) (*p* = 0.129).	NR
[107]	Ping et al., 2022	Retrospective	ICSI infants from NOA patients (84)	ICSI infants from extremely OZ (163) Severe OZ (174) Mild OZ (148) OA (155) Normozoospermia (210)	Miscarriage rate		NOA patients had a lower miscarriage rate (2/84; 3.3%), but the difference was not statistically significant (*p* = 0.44). Extremely OZ (9/163; 7.2%); Severe OZ (5/174; 3.9%); Mild OZ (7/148; 7.5%); OA (3/155; 2.7%); Normozoospermia (10/210; 6.2%).
[108]	Xu et al., 2023	Retrospective	ICSI cycles from NOA patients (158) using testicular fresh sperm	ICSI cycles from OA patients (435) and oligoasthenozoospermia patients (92) using fresh testicular sperm	ICSI outcomes	From 158 TESE-ICSI cycles performed in NOA patients, the clinical pregnancy rate was 66.5% (105/158), and the live birth rate was 59.5% (94/158).	There were no significant differences between the three groups in terms of biochemical pregnancy rate, clinical pregnancy rate, live birth rate, or abortion rate. Miscarriage rates: Oligoasthenozoospermia 5.43% (5/92); OA 6.9% (30/435); NOA 5.06% (8/158) (*p* = NS).
[78]	Elzeiny et al., 2024	Retrospective	NOA-ICSI cycles (63)	NR	Neonatal outcomes	From 63 NOA-ICSI cycles, there were 39 clinical pregnancies, and 2 miscarriages reported.	NR

ICSI, intracytoplasmic sperm injection; KS, Klinefelter syndrome; micro-TESE, microdissection testicular sperm extraction; NOA, non-obstructive azoospermia; NR, not reported; NS: non-significant; OA, obstructive azoospermia; OAT, oligoasthenoteratozoospermia; OZ, oligozoospermia; TESE, testicular sperm extraction.

### 4.2. Chromosomal Abnormalities

A total of 17 studies examined the frequency of chromosomal abnormalities either in the retrieved sperm from men with NOA or the resulting embryos following preimplantation genetic testing (PGT) [110,111,112,113,114,115,116,117,118,119,120,121,122,123,124,125,126]. Nine of these studies were prospective, while the remaining were retrospective (Table 2). Most studies explored chromosomal abnormalities in men with NOA and OA, including a comparator group of men with normal semen quality. A few studies also included males with various sperm abnormalities [117,126]. The outcome measures focused on various chromosomal abnormalities such as aneuploidy, disomy, and nullisomy for different chromosomes, including sex and various autosomes. In 14 studies, these aberrations were assessed in the retrieved testicular sperm [110,111,112,113,114,115,116,118,119,120,122,123,124,126], while 3 studies focused on the quality of the obtained embryos following ICSI [117,121,125]. Overall, the retrieved sperm of NOA patients exhibited higher rates of chromosomal abnormalities (aneuploidy, disomy, diploidy) compared to OA patients and/or fertile controls [111,112,114,115,116,119,120]. Compared to NOA patients with normal karyotype, KS patients appear to have a higher rate of sperm aneuploidy (5.3% vs. 4.0%; *p* = 0.0089) and chromosome 18 abnormalities (1.43% vs. 1.19%, *p* < 0.001) [123]. Additionally, fresh and frozen-thawed testicular sperm samplers showed similar incidences of chromosomal abnormalities for chromosomes 13, 18, 21, and sex chromosomes in NOA patients [118].

Two studies addressed the impact of the sperm source (testicular vs. ejaculate) regarding the incidence of chromosomal abnormalities using a control group of non-azoospermic donors [122,126]. Thus, Rodrigo et al. [122] observed a higher incidence of chromosomal abnormalities in the testicular sperm of NOA patients as well as fertile donors compared to ejaculated sperm from the same donors. However, no differences in the percentage of genetically abnormal sperm were observed when surgically retrieved sperm from azoospermic patients were compared with testicular sperm from fertile donors [122]. Conversely, Cheung et al. observed higher rates of sperm aneuploidy in the ejaculated sperm of non-azoospermic infertile men (11.1%) compared to epididymal sperm from OA men (1.8%) and testicular sperm from NOA men (1.5%) (*p* < 0.0001) [126].

### 4.3. Congenital Malformations

Congenital malformations in the offspring of NOA biological fathers were reported by 19 studies [62,78,80,81,106,107,127,128,129,130,131,132,133,134,135,136,137,138,139]. Most of these retrospective studies compared the outcome between patients with NOA and OA. Fewer studies included additional control groups of men with variable sperm abnormalities or normal fertile controls [106,107,129,137]. Across the studies, the reported incidence of congenital malformations following ICSI was extremely low in all the studied groups, preventing meaningful statistical analyses (Table 3).

The types of congenital malformations observed included major anomalies like polydactyly, cleft lip and palate, cryptorchidism, hypospadias, and cardiovascular defects, as well as minor anomalies such as bilateral inguinal hernia and open ductus arteriosus. Lan et al. [62] and Zhang et al. [81] compared congenital malformations in the offspring of NOA patients secondary to various etiologies, including KS, AZFc, cryptorchidism, orchitis, and idiopathic causes. Lan et al. [62] observed the highest rate of preterm birth (50%) in patients with AZFc microdeletion (*p* < 0.05). Zhang et al. [81] reported three cases of pre-term birth across all the study groups, all of which belonged to patients with KS. However, no differences in birth defects were observed between the use of fresh or frozen-thawed sperm from NOA males [80,134]. In our study, referenced in the previous section [106], a total of two deliveries (out of 48 infants delivered) involved either a perinatal death or a malformation (cleft lip and palate) in the group of men with NOA, resulting in an overall adverse neonatal outcome of 4.1%, not statistically different from the OA and donor sperm groups.

### 4.4. Psychological and Neurological Development

Two studies by Tsai et al. assessed the psychological and neurological development of offspring from NOA patients born through TESE-ICSI [136,140] (Table 4). In their first study, the authors compared perinatal outcomes and development of children assessed at the age of 1–7 years among the offspring of males with NOA and OA, comparing them to offspring of men with oligoasthenoteratozoospermia [140]. The authors did not observe any differences in children’s psychomotor or intellectual development across the studied groups.

**Table 3:** Studies evaluating congenital malformations in ICSI infants from NOA fathers.

Reference #	Study	Design	Study Group (n)	Control Group (n)	Outcome Measures	Findings	
						NOA Infants	NOA-Infants vs. Comparator
[127]	Palermo et al., 1999	Retrospective	ICSI infants from NOA patients (22)	ICSI infants from OA patients (158)	Congenital malformation	Of 22 NOA-ICSI infants, only one child was born with a malformation (4.5%).	The incidence of congenital malformation did not vary according to the sperm origin or cause of azoospermia: OA: 1.3%; NOA: 4.5%.
[128]	Scholtes et al., 1999	Retrospective	ICSI infants (160) NOA (116)	OA (44)	Congenital malformation	From 36 live births, there was 1 case of congenital malformation (not specified).	NR
[129]	Ludwig et al., 2003	Retrospective	ICSI infants from: NOA patients (86); OA patients (68)	ICSI infants from OAT patients (1980)	Major congenital malformation	Of 112 NOA-ICSI infants, eight children were born with a major malformation (7.1%).	There were no differences between groups in major malformation: OAT: 8.7%; OA: 8.4%; NOA: 7.1%.
[130]	Vernaeve et al., 2003	Retrospective	ICSI infants with testicular sperm from NOA patients (83): Fresh sperm (72); Frozen-thawed sperm (11)	ICSI infants with testicular sperm from OA patients (216): Fresh sperm (189) Frozen-thawed sperm (27)	Congenital malformation	NOA-ICSI infants malformations: Major malformation: Polydactyly pre-axial fingers Bilateral cleft lip Minor malformation: Bilateral inguinal hernia in premature child	Among live-born children, major malformations rates were: NOA: 4% (2/54); OA: 3% (5/188); (RR: 1.4, 95% CI: 0.19–7.8). The rates of minor malformations were: NOA: 2% (1/54); OA: 4% (8/188) (RR: 0.4, 95% CI: 0.02–3.27).
[131]	Vernaeve et al., 2004	Retrospective	TESE-ICSI cycles performed in NOA patients (156): Orchidopexy (64); Unexplained (92)	NR	Congenital malformation	In the 15 live-born children in the orchidopexy group, one major (lipomeningocoele) and one minor (open ductus arteriosus) malformation were observed. No malformations were observed in the live-born children in the unexplained group.	NR
[132]	Fedder et al., 2007	Retrospective	ICSI infants from NOA patients (76)	ICSI infants from OA patients (282)	Congenital malformation	A total of 76 children were born to NOA patients, and none had any malformations.	There were no differences in congenital malformation among the groups: OA: 4.0%; NOA: 0%.
[133]	Belva et al., 2011	Prospective	ICSI infants from NOA patients (193)	ICSI infants from OA patients (474)	Congenital malformation	Of 168 NOA-ICSI infants, seven children were born with major malformation, of which three were genital malformation (cryptorchid testes and hypospadias).	There were no differences in congenital malformation among the groups: OA: 5.2%; NOA: 4.2%.
[134]	Tavukcuoglu et al., 2013	Retrospective	Micro-TESE-ICSI cycles from NOA patients (82): Fresh sperm (43) Frozen-thawed sperm (39)	NR	ICSI outcomes	There were no statistically significant differences in embryo quality, clinical pregnancy, live birth, and miscarriage rates when using fresh and frozen-thawed sperm in ICSI cycles from NOA patients. No congenital anomalies or major malformations were noted in both groups.	NR
[135]	Oron et al., 2014	Retrospective	ICSI infants (108) NOA (54)	OTA (54)	Fetal malformation	From 39 live births, there were 3 cases (7.6%) of fetal malformation (bilateral inguinal hernia, multiple malformations, polydactyl).	The fetal malformation rates were similar between the groups (*p* = NS).
[106]	Esteves et al., 2014	Retrospective	ICSI infants from testicular sperm by micro-TESE of NOA patients (151 cycles) ICSI infants from testicular sperm by TESA of OA patients (146 cycles)	ICSI infants from ejaculated sperm of donors (40 cycles)	Outcomes of neonates	In the group of NOA patients, two deliveries involved either perinatal death (2.1%, 1/48) or a malformation (cleft lip and palate) (2.1%, 1/48), leading to an overall adverse neonate outcome rate of 4.1%.	Among 24 neonates born from donor sperm, there were no cases of congenital malformations. By contrast, among 65 neonates born to OA patients, there was one case of perinatal death (1.5%, 1/65) and one case of malformation (1.5%, 1/65). However, the rates of congenital malformations and perinatal deaths did not significantly differ between the three groups (*p* = NS).
[136]	Tsai et al., 2015	Retrospective	ICSI infants (154); NOA (87)	OA (67)	Clinical outcomes	Only 1 case of heart minor anomaly and 1 case of heart major anomaly were reported in the NOA group. There were no musculoskeletal or urogenital system anomalies.	Neonatal outcomes were similar in the two groups, with comparable minor congenital anomalies (heart, musculoskeletal system, and urogenital system) and major congenital anomalies (heart major anomalies).
[137]	Yu et al., 2018	Retrospective	ICSI infants (225); NOA (44)	OA (126) Donor sperm (62)	Clinical outcomes	No baby was stillborn or had malformations in the NOA group.	One baby (2.2%) was stillborn due to megabladder in the donor sperm group. In the OA group, two pairs of twins (3.8%) died shortly after their premature birth (gestational age of 24 weeks and 28 weeks, respectively), and one baby (1.0%) had hypospadia. Live birth rates were significantly lower in the NOA group than in the donor sperm group (24.6% vs. 41.3%, *p* = 0.04) but not significantly lower than in the OA group (*p* = NS). Live birth rates were similar between the OA group and the donor sperm group.
[81]	Zhang et al., 2021	Retrospective	ICSI infants from NOA patients (769): KS (284)—125 cycles AZFc microdeletion (91)—64 cycles Cryptorchidism (52)—39 cycles Orchitis (23)—23 cycles Idiopathic (319)—96 cycles	NR	Congenital defects	No difference was found in birth defects among all groups (*p* > 0.05). Only three cases of birth defects were reported, all in the KS group.	NR
[79]	Zhang et al., 2021	Retrospective	ICSI infants from NOA patients (338): Fresh sperm (222) Cryopreserved sperm (116)	NR	Congenital defects	No difference was found in birth defects among the group with fresh or cryopreserved sperm. Only three cases were reported in the group with fresh sperm (3/108, 2.8%), while no case was reported in the group with cryopreserved sperm (0/40, 0%).	NR
[63]	Lan et al., 2022	Retrospective	ICSI infants from NOA patients (968)	NR	Clinical outcomes	From 140 live-birth deliveries, the birth defects rate was 1.43% (one case with cardiovascular malformation and the other with a cleft lip and palate). Singleton newborns of the frozen sperm group had higher height compared to the fresh sperm group (49.84 ± 2.04 cm vs. 48.50 ± 3.03 cm, *p* < 0.05). Among different etiologies of NOA, the highest rate of premature birth (50%) was observed in patients with Y chromosome AZFc microdeletions (*p* < 0.05).	NR
[107]	Ping et al., 2022	Retrospective cohort	ICSI infants from NOA patients (84)	ICSI infants from extremely OZ (163) Severe OZ (174) Mild OZ (148) OA (155) Normozoospermia (210)	Congenital defects	There were only two cases of major birth defects (both were patent foramen ovale): one in the NOA group (1/84; 1.6%) and the other in the normozoospermic group (1/210; 0.6%) (*p* = 0.34).	NR
[138]	Romano et al., 2023	Retrospective	ICSI infants from NOA patients (260) 446 COS cycles	ICSI infants from OA patients (290) 620 COS cycles	Congenital defects	Neonatal outcomes were similar in the two groups, with comparable gestational age and birth weight for single or twin pregnancies (*p* = 0.32).	OA: 7 cases of congenital defects (2 cases of persistently patent arterial duct of Botallo, hypospadias, bilateral clubfoot, right-side hemispondyl, cryptorchidism, and pharyngeal defects). NOA: 2 cases of congenital defects (intraventricular defect and hypospadias).
[139]	Zhang et al., 2023	Retrospective	ICSI infants from NOA (235) ICSI cycles with immotile sperm injection (101) with AOA	ICSI cycles with motile sperm injection (230) AOA (129) Non-AOA (101)	Clinical outcomes	From 141 live-birth deliveries, there were no early neonatal deaths or birth defect cases.	Neonatal outcomes, including singleton and twin birth rate, baby’s birth weight, and baby’s body length, were comparable among the three groups.
[78]	Elzeiny et al., 2024	Retrospective	ICSI infants from NOA patients (108)	NR	Neonatal outcomes	From 63 couples who started ICSI, a total of 47 live offspring with no neonatal deaths or defects were reported.	NR

AOA, artificial oocyte activation; ART, assisted reproductive technology; CI, confidence interval; COS, controlled ovarian stimulation; ICSI, intracytoplasmic sperm injection; IVF, conventional in vitro fertilization; KS, Klinefelter syndrome; NC, naturally conceived; NR, not reported; NS: non-significant; OTA, oligoteratoasthenospermia; OZ, oligozoospermia; RR, relative risk.

In their second study, the authors compared men with NOA to OA and assessed their offspring’s feeding and sleeping behavior, posture, coordination, memory, and problem-solving, language, and socialization skills. The results revealed normal health for the children conceived through ICSI, with none showing handicaps in psychomotor or intellectual development [136].

Based on the above-mentioned studies, the following can be concluded: sperm from NOA males and embryos obtained after ICSI with testicular sperm from NOA males may have an increased likelihood of chromosomal abnormalities. However, once fertilization and implantation have occurred, the current evidence does not suggest a higher risk of miscarriage compared to what is observed in infertile men or fertile donors. Moreover, a low risk of congenital malformations and adverse neurodevelopmental features has been reported in the identified studies, further supporting the safety of ICSI using testicular sperm from NOA patients. Nevertheless, data still remain limited, which calls for continuous monitoring. Notably, despite our extensive literature search, we did not identify studies reporting on epigenetic disorders, infertility, cancer, and cardiometabolic profiles of children conceived using testicular sperm from NOA males. The frequency of these conditions in the offspring of NOA males has yet to be determined. Finally, a limitation of the collected studies is that very few cases for certain outcomes have been reported, making it difficult to generalize the results.

## 5. Managing Patient Expectations

### 5.1. Genetic Considerations and Counseling

Counseling plays a critical role when managing infertile couples, especially when the male partner is diagnosed with NOA. This diagnosis significantly impacts the couple’s mental health, making it essential for clinicians to address uncertainties and provide realistic expectations about outcomes from procedures like testicular sperm retrieval and ICSI [141]. Infertility, particularly severe spermatogenic dysfunction, is linked to several health risks. For example, infertile men face a 20-fold higher risk of testicular cancer compared to their fertile counterparts of the same age and race [142]. Additionally, risks for other cancers, such as colorectal cancer, melanoma, and prostate cancer, are also increased [143]. Counseling should include discussions about the patient’s overall health and advice on screening for these associated conditions.

Counseling becomes particularly vital when genetic abnormalities are detected in NOA patients, as in many instances, the diagnosis can impose additional risks on the patients and the offspring.

Disorders like KS not only lead to primary hypogonadism but also increase the risk of metabolic syndrome, diabetes, mitral valve prolapses, and breast cancer [144]. Discussions regarding health consequences and potential genetic risks to the offspring of NOA patients with chromosomal abnormalities should also be covered. Conditions like KS are associated with a higher risk of sperm aneuploidy, and while the majority of children are born healthy with a normal chromosomal makeup, there have been instances of 47,XXY pregnancies [145,146].

Moreover, male offspring of patients with Y chromosome microdeletions will undoubtedly inherit the same or have even worse deletions compared to their fathers [147].

Furthermore, patients with Robertsonian or reciprocal translocations or chromosome inversions are at an increased risk of producing gametes with unbalanced chromosomal contents, potentially leading to infertility, miscarriage, or offspring with congenital abnormalities [148,149].

Genetic counseling can help these couples understand their reproductive options, including the use of pre-implantation genetic testing (PGT) to identify and select embryos with balanced chromosomes or even perform sex selection to avoid gender-specific genetic risks.

Equally important is the integration of psychologic support with genetic counseling in recognition of the mental health burdens that are often experienced by couples with NOA. By providing comprehensive, multidisciplinary care, healthcare providers can enhance the well-being, treatment adherence, and overall outcomes for these couples.

### 5.2. Reducing the Risk for Offspring: Pros and Cons of PGT-A

PGT is employed within the context of IVF to screen embryos for genetic abnormalities before transfer (Figure 2) [150]. It has been indicated for couples with advanced maternal age, a history of recurrent pregnancy loss or failed implantation, and those with known genetic abnormalities [150]. However, PGT can also be performed for gender selection to avoid certain sex-linked diseases. Genetic testing is performed on DNA obtained from biopsied embryos, typically at the blastocyst stage. Three different types of PGT exist: PGT-A for aneuploidy, PGT-M for single-gene disorders, and PGT-SR for structural rearrangements [150]. The procedure refines embryo selection before implantation, aiming to increase the chances of a healthy child and reduce the risk of genetic diseases being passed on to the offspring [151].

The scientific community supports the use of PGT-M and PGT-SR [152]; however, PGT-A remains controversial despite its routine application in numerous fertility centers worldwide [153]. While it can reduce the risk of aneuploidy and potentially increase birth rates, drawbacks include the need for additional resources and up to eight cumulative hours of labor from the embryology team for each biopsy case [153]. Additionally, not all embryos reach the blastocyst stage necessary for biopsy—some of these could have led to healthy births if transferred earlier [154]. Moreover, mosaicism, where embryos contain genetically diverse cells, is more common in preimplantation stages than previously expected. This complexity poses challenges in understanding and applying PGT-A, as a mosaic embryo might still develop into a healthy baby [153]. Studies suggest that mosaic embryos might self-correct during development, implying that a biopsy may not always represent the embryo’s overall genetic makeup, potentially misleading PGT-A results [155].

Although the main advantage of PGT is its ability to screen for specific genetic disorders before embryo implantation, potentially reducing the risk of hereditary diseases in the child, it does have limitations and ethical concerns. For instance, it cannot guarantee that the baby will be free from all genetic abnormalities [156]. Significant variability in how PGT is regulated and used across different clinics also affects its reliability and outcomes. Lastly, the cost and the lack of comprehensive insurance coverage for PGT can make it inaccessible for many couples [157]. The need for more rigorous clinical validation and better patient education on PGT is emphasized to ensure it is used appropriately and effectively.

Lastly, with ICSI and advanced genetic testing, ethical considerations are paramount. Patients must be fully informed about the potential risks, benefits, and limitations of these procedures. Informed consent should involve a detailed discussion of the possible outcomes, including the ethical implications of selecting embryos based on genetic information. It is essential to ensure that patients understand the complexity of genetic data, the uncertainty of some results, and the potential emotional and psychological impacts. Additionally, transparency about the success rates and potential long-term effects on offspring should be provided to allow patients to make well-informed decisions.

## 6. Future Directions

Several emerging technologies for the treatment of NOA focus on enhancing the reproductive possibilities for affected individuals through advancements in sperm retrieval efficacy, accuracy, and the use of cell-based therapies.

### 6.1. Advances in Testicular Sperm Retrieval

The success of microsurgical TESE largely depends on the surgeon’s skill in detecting seminiferous tubules that contain spermatozoa and the embryologist’s ability to find sperm from the retrieved specimens. This challenge is directly related to the severity of the spermatogenic dysfunction. Surgeons evaluate the seminiferous tubules using a subjective method that assesses their size and opacity with an operating microscope.

Various innovative technologies have been explored and might be integrated into microsurgical TESE procedures to facilitate sperm selection and enhance the likelihood of successful sperm retrieval. Examples include multiphoton microscopy (MPM), Raman spectroscopy (RS), full-field optical coherence tomography (FFOCT), and ultrasonography (US).

MPM utilizes a near-infrared femtosecond pulsed laser that penetrates deep into testicular tissues, enabling detailed imaging of the lumina of seminiferous tubules through optical sectioning [158]. By providing high-resolution images in real-time, MPM allows for specifically targeting sperm-containing tubules and limits extensive dissection of testicular tissue. At the same time, MPM has shown potential in improving the detection of sperm within seminiferous tubules [159]; however, concerns regarding thermal and nonlinear damage to DNA exist, which could lead to genetic anomalies in gametes destined for IVF. Although studies in rodent models have reported minimal phototoxicity [160], these results need confirmation in human studies to ensure the safety and efficacy of this technique.

RS is a laser-based, label-free probe that operates on the principle of inelastic scattering from molecular vibrations. It takes advantage of the unique molecular fingerprints of different tissues to convert biochemical information into a distinctive Raman spectrum [161]. RS has been shown to have a high degree of sensitivity (96%) and specificity (100%) in detecting spermatogenesis in rat models with SCO histology [162]. The precision of this adjuvant method suggests that RS-guided microsurgical TESE could significantly enhance sperm retrieval rates. Although RS is non-invasive and non-destructive, the safety of this laser-based method still requires evaluation in human studies.

FFOCT is a technique that utilizes white-light interference microscopy principles to generate high-resolution images of unprocessed, unstained tissue [163]. Unlike MPM and RS, it uses a low-power, 150 W halogen lamp, which is inherently safer and reduces the likelihood of causing thermal DNA damage. In a pilot study on rodents, FFOCT demonstrated its ability to differentiate between tubules with and without spermatogenesis [164]. Lastly, US has also been explored as a non-invasive, readily available technique to assess spermatogenesis during testicular sperm retrieval. Interest in US as an adjunct procedure during testicular sperm retrieval is based on findings suggesting a direct relationship between areas of active spermatogenesis and increased testicular blood perfusion. Until now, intraoperative US-guided sperm retrieval, as well as preoperative contrast-enhanced US, have been utilized [165,166], but overall, its use as a screening method for sperm detection has a low sensitivity and a high specificity, indicating that US may be used to deselect areas of absent spermatogenesis [167].

Finally, the recent integration of artificial intelligence (AI) and machine learning (ML) technologies in the field of reproductive medicine may potentially enhance ART outcomes for men with NOA. Advanced imaging techniques combined with AI may be utilized during surgical sperm retrieval or in processing the retrieved tissue samples to improve sperm discovery and selection [168].

The technologies discussed above are still evolving, and further studies are needed before they can be fully integrated into clinical practice.

### 6.2. Stem Cell Therapy

Studies on cell-based therapies for NOA have focused on two primary functions: their ability to regenerate tissue and/or their paracrine or anti-inflammatory effects. Several cell types have been investigated for these indications, including spermatogonial stem cells (SSCs), embryonic stem cells (ESCs), very small embryonic stem cells (VSELs), and mesenchymal stem cells (MSCs). SSCs can self-renew and progress into progenitor spermatogonia, which may eventually differentiate into spermatozoa [169]. SSCs are mainly investigated for fertility preservation in prepubertal cancer patients before receiving anti-neoplastic therapy [170]. Nonetheless, SSCs can also be isolated from adult NOA males [171]. Studies conducted in vitro and through various models of autografting, allografting, and xenografting—involving both animal and hybrid human–animal systems—have achieved some functional success, including producing fertile offspring in mice [172,173]. These findings provide a basis for cautious optimism, as concerns about the potential for carcinogenesis and genetic/epigenetic alterations in the offspring exist. These issues must be thoroughly addressed before SSC transplantation can be safely and effectively translated into human clinical practice.

Among the various other cell types, MSCs are commonly investigated due to their proliferative, immunomodulatory, anti-inflammatory, and anti-apoptotic properties, making them suitable for inducing spermatogenesis and treating azoospermia [174]. MSCs can be obtained from various tissues, including adipose tissue, peripheral blood, and bone marrow. Numerous studies, both in vitro and in vivo (reviewed by [175]), have demonstrated that MSCs not only have the potential to differentiate into germ cells, but can also enhance the tubular microenvironment of the testes through paracrine effects. In a recent human study, a total of 87 NOA males received a single intra-testicular injection of MSCs derived from their bone marrow [176]. Sperm was observed in the ejaculate of 20.7% of patients, showing promise in fertility restoration. Notably, most of them had a SCO histology (61.1%), and none of the responders had any chromosomal abnormalities.

While these technologies may offer hope for NOA men with failed sperm retrievals, they are still evolving, and the reported success rates are mostly anecdotal. Further studies are needed before they can be integrated into clinical practice.

### 6.3. Gene Editing Technologies

The recent application of gene-editing technologies, particularly CRISPR (Clustered Regularly Interspaced Short Palindromic Repeats), represents a revolutionary yet complex frontier in reproductive medicine. The CRISPR-Cas9 technology allows for precise genome editing by targeting specific DNA sequences and making controlled modifications. Its application may include disabling or removing harmful genes, correcting specific mutations, and inserting new genes to alter particular genomic functions [177]. In 2017, the first therapeutic germline intervention using CRISPR-Cas9 was reported, where researchers corrected the genetic defects of zygotes resulting from the microinjection of sperm with the MYBPC3 mutation, which predisposes the offspring to develop hypertrophic cardiomyopathy [178]. This technology may offer some hope for men with genetic NOA. However, it should be approached with caution, as it is accompanied by complex ethical and regulatory challenges [179].

## 7. Conclusions

This review provides a comprehensive analysis of the efficacy and outcomes of ICSI in patients with NOA. The key takeaways emphasize that ICSI, coupled with testicular sperm retrieval techniques such as microsurgical TESE, offers a viable reproductive option for men who were previously deemed sterile. While ICSI outcomes for NOA patients are associated with lower fertilization, clinical pregnancy, and live birth rates compared to OA and non-azoospermic patients, successful pregnancies and live births are achievable. This underscores the potential of ICSI to enable biological parenthood for men with severe spermatogenic dysfunction. The role of ICSI in treating NOA is essential, providing hope where conventional methods fail. Factors such as the sperm retrieval technique, histopathological patterns, hormone-based therapy prior to sperm retrieval, and varicocelectomy in selected cases, as well as the age of the female partner, influence ICSI outcomes in the NOA scenario. The success of microsurgical TESE in identifying sperm within seminiferous tubules has been particularly highlighted, offering better retrieval rates with minimal tissue damage. We encourage azoospermic patients to seek specialized reproductive care, as advancements in laboratory techniques of testicular sperm handling and microsurgical TESE provide hope for achieving biological parenthood. Counseling and managing patient expectations are crucial, as personalized treatment plans can significantly enhance the likelihood of success. Genetic counseling should also be emphasized to address potential risks and ensure informed decision making. Furthermore, emerging technologies such as multiphoton microscopy, Raman spectroscopy, full-field optical coherence tomography, and cell-based therapies like stem cell transplantation show potential for future improvements when handling NOA patients.

## 8. Review Criteria

An extensive search of studies examining the relationship between non-obstructive azoospermia and intracytoplasmic sperm injection was performed using PubMed and MEDLINE. The start and end dates for the search were January 1997 and May 2024, respectively. The overall strategy for study identification and data extraction was based on the following key words: “assisted reproductive technology”, “intracytoplasmic sperm injection”, “male infertility”, “non-obstructive azoospermia”, “pregnancy outcomes”, and “children”, with the filters “humans” and “English language”. Using the mentioned criteria, 71 relevant articles were identified. Data only published in conference or meeting proceedings, websites, or books were not included. Citations dated outside the search dates were only included if they provided conceptual content.

## Figures and Tables

**Table 2:** Studies evaluating chromosomal aberrations in embryos of couples undergoing ICSI with testicular sperm from NOA patients.

Reference #	Study	Design	Study Group (n)	Control Group (n)	Outcome Measures	Findings	
						NOA-ICSI Infants	NOA-ICSI Infants vs. Comparator
[110]	Martin et al., 2000	Prospective	NOA patients (3)	Ejaculated sperm from fertile donors (18)	Sperm numerical abnormalities for chromosomes 13, 21, X, and Y, as well as the proportion of X- and Y-chromosome-bearing spermatozoa and diploidy	The frequency of disomy for chromosome 13, 21, and XY disomy was elevated but without statistical significance.	The only statistically significant difference between the infertile patients and control donors was for the proportion of YY disomy in which NOA patients had 0% compared to 0.06% in controls (*p* < 0.001)
[111]	Bernardini et al., 2000	Retrospective	NOA patients (3) OA patients (6) Patients with severe OAT (22) Patients with unexplained infertility (3)	Healthy donors (10)	Sperm aneuploidy for chromosomes X, Y, 1, and 17	The frequency of spermatozoa aneuploidy, diploidy, and nullisomy for chromosomes 1 and 17 was significantly higher in NOA patients than in the other groups (unexplained infertility, OAT, and OA; *p* < 0.00001). The frequency of sex chromosome DNA-ploidy and nullisomy were also higher in NOA patients than in the other groups (unexplained infertility, OAT, and OA; *p* < 0.00001).	The frequency of spermatozoa aneuploidy, diploidy, and nullisomy for chromosomes 1 and 17 was significantly higher in NOA patients than in controls (*p* < 0.01). The frequency of sex chromosome DNA-ploidy and nullisomy were also higher in NOA patients than in controls (*p* < 0.01).
[112]	Levron et al., 2001	Retrospective	Testicular spermatozoa (9): NOA patients Testicular spermatozoa (10): OA patients Ejaculated spermatozoa (9): Oligoasthenoteratospermia patients	Ejaculated spermatozoa (6) from normal fertile donors	Sperm numerical abnormalities for chromosomes 18, X, and Y	The aneuploidy rates among the sperm were: 19.6% (30/153) of NOA patients; 8.2% (30/367) of OA patients; 13.0% (228/1751) of severe OAT; 1.6% (8/500) of controls. The disomy rates among groups were: 7.8% (12/153) in NOA patients; 4.9% (18/367) in OA patients; 6.2% (109/1751) in severe OAT; 1% (5/500) in controls. The disomy rates in groups of NOA, OA, and OAT patients were significantly higher than the controls (*p* < 0.001). In addition, the disomy rate was significantly higher in NOA and OAT patients than OA patients (*p* < 0.01).	NR
[113]	Burrello et al., 2002	Prospective	Testicular spermatozoa (6): NOA patients Epididymal spermatozoa (10): OA patients	Ejaculated spermatozoa (14) from healthy men	Sperm numerical abnormalities for chromosomes 8, 12, 18, X and Y	The frequency of total sex chromosome disomy was higher in testicular sperm (2.07%; range: 1.02 ± 6.25) than ejaculated sperm (0.43%; 0 ± 0.90%; *p* < 0.05), but not statistically different than epididymal sperm (1.38%; 0.75 ± 5.76); the frequency of autosome nullisomy was comparable among groups. The frequency of total autosome disomy (chromosomes 8, 12, and 18) was higher in epididymal (1.2%; 0 ± 4.09%) and testicular (2.23%; range: 0.96 ± 17.4%) spermatozoa compared to those in ejaculated spermatozoa (0.46%; 0.15 ± 0.65%; *p* < 0.05); the frequency of autosome nullisomy was comparable among groups.	The frequencies of sex chromosome and autosomes disomy were statistically higher in both testicular and epididymal spermatozoa compared to ejaculated spermatozoa.
[114]	Mateizel et al., 2002	Retrospective	Testicular spermatozoa (17): NOA patients	Testicular spermatozoa (26) from men with normal spermatogenesis	Sperm numerical abnormalities for chromosomes 18, X, and Y	There was no significant difference in numeric chromosomal abnormalities among the groups (8.2% vs. 5.6%, *p* = 0.076). Similarly, no differences were found in total disomy (2.5% vs. 3.7%, *p* > 0.05).	The proportion of sex chromosome aneuploidy was 5.8% in NOA patients and 4.5% in controls (*p* > 0.05). However, there was a significantly higher frequency of aneuploidy for chromosome 18 in NOA patients (3.2%) than the control group (1.3%; *p* = 0.016). The frequency of sex chromosome disomy was similar among groups (2.2% vs. 2.4%, *p* > 0.05). However, a higher frequency of disomy for chromosome 18 was observed in NOA patients (1.3%) than in the control group (0.3%; *p* = 0.05). Neither sex chromosome nullisomy frequency nor chromosome 18 nullisomy frequency were different between NOA patients and control.
[115]	Palermo et al., 2002	Prospective	Testicular spermatozoa (5): NOA patients Epididymal spermatozoa (8): OA patients	Ejaculated spermatozoa (14) Healthy men	Sperm numerical abnormalities	The sperm aneuploidy rate was 11.4% in NOA men, 1.8% in OA patients, and 1.5% in ejaculate controls.	The incidence of chromosomal abnormalities in the NOA patients was significantly higher than in the OA and control groups (*p* = 0.0001); the most predominant abnormality in NOA men was sex chromosome disomy (37.5%), followed by nullisomy (32.1%).
[116]	Martin et al., 2003	Prospective	NOA patients (6)	Ejaculated sperm from fertile donors (18)	Sperm numerical abnormalities for chromosomes X and Y	The frequency of sex chromosomal disomy for XY, YY, and total sex chromosome disomy and diploidy was higher compared with control donors, but only YY disomy reached statistical significance (*p* = 0.02).	One NOA patient had a frequency of 3.8% XY disomy and 4.3% diploidy, 13-fold and 7-fold higher than control donors, respectively.
[117]	Silber et al., 2003	Retrospective	Embryos derived from 19 TESE-ICSI cycles of NOA patients (100)	Embryos derived from 111 cycles of ICSI with ejaculated sperm from oligozoospermic patients (830)	Embryo numerical abnormalities for chromosomes 13, 15, 16, 18, 21, 22, X and Y	The rates of normal embryos were higher in the oligozoospermia-ICSI group than TESE-ICSI group (41.8% vs. 22%, *p* < 0.001). In addition, the rates of mosaic embryos were higher in the TESE-ICSI group than in the oligozoospermia-ICSI group (53% vs. 26.5%, *p* < 0.001). The rates of aneuploidy per chromosome were similar in both groups, including 2.9% and 1% rates of sex chromosome aneuploidy in oligospermia-ICSI and TESE-ICSI groups.	NR
[118]	Rodrigo et al., 2004	Prospective	NOA patients (13) OA patients: Testicular sperm (7) Epididymal sperm (2)	Normozoospermic patient: Ejaculated sperm (5)	Sperm numerical abnormalities for chromosomes 13,18, and 21 and sex chromosomes X and Y	Fresh and frozen-thawed testicular sperm samplers showed similar incidences of chromosomal abnormalities for chromosomes 13, 18, 21, and sex chromosomes in NOA patients.	Testicular samples from NOA patients showed significantly higher rates of diploidy (*p* < 0.0001) and disomy chromosomes 13 (*p* < 0.0001) and 21 (*p* < 0.001) and for sex chromosomes (*p* < 0.0001) than those of the control group. The incidence of diploidy and disomy for sex chromosomes rates was higher in the testicular samples from NOA patients when compared to testicular samples from OA patients.
[119]	Ma et al., 2006	Retrospective	Testicular sperm from NOA patients (3)	Testicular sperm from OA patient (1)	Sperm numerical abnormalities	The overall aneuploidy, sex aneuploidy, sex disomy, and sex nulisomy of the NOA patients were higher than those of control (*p* < 0.05).	NR
[120]	Sun et al., 2008	Prospective	Testicular sperm from NOA patients (7)	Ejaculated sperm from a control group after vasectomy reversal (6)	Sperm numerical abnormalities for chromosomes 9, 21 and sex chromosomes X and Y	The frequency of disomy for chromosome 21 (*p* = 0.001), XX (0.004), and YY (0.04) was significantly elevated in NOA patients compared with controls.	NR
[121]	Magli et al., 2009	Retrospective	OAT men (134 cycles, of which 76 were severe OAT), OA men (29 cycles), and NOA men (27 cycles)	Normozoospermic men (105 cycles)	Embryo numerical abnormalities for chromosomes 13, 15, 16, 17, 18, 21, 22, X and Y	The frequency of abnormal embryos was significantly lower in normozoospermic patients (55%) than in OA (62%, *p* < 0.025) and NOA (69%, *p* < 0.005).	NR
[122]	Rodrigo et al., 2011	Prospective	Testicular sperm from: OA patients (16); NOA patients (19)	Ejaculated sperm from fertile donors (10) Testicular sperm from fertile donors (10)	Sperm numerical abnormalities for chromosomes 13, 18, 21, X and Y; Implantation and ongoing pregnancy rates in ICSI cycles	Testicular sperm from fertile donors showed a higher incidence of diploidy (0.27% vs. 0.10%; *p* < 0.0001) and disomy for chromosomes 13 (0.16% vs. 0.07%; *p* < 0.05) and 21 (0.25% vs. 0.12%; *p* < 0.01), and sex chromosomes (0.34% vs. 0.21%; *p* < 0.05) than ejaculated sperm from fertile donors. Sperm chromosomal abnormalities were higher in surgically retrieved gametes from azoospermic men (12.5% in OA and 68.4% in NOA) than in ejaculated sperm from fertile donors. No differences in the percentage of genetically abnormal sperm were observed when surgically retrieved sperm from azoospermic patients were compared with testicular sperm from fertile donors. ICSI reproductive outcomes in NOA patients resulted in a significantly lower fertilization rate and poorer embryo quality than in OA patients. The ongoing pregnancy rate per ICSI cycle was lower for NOA than OA patients (21.4% vs. 38.1%).	NR
[123]	Vialard et al., 2012	Retrospective	Testicular sperm from: KS patients (10) NOA patients with normal karyotype (19) OA patients (22)	Normal sperm analysis (11)	Sperm numerical abnormalities for chromosomes 18, X, and Y by FISH	The aneuploidy rates were higher in KS patients (5.3%) than in NOA patients with normal karyotypes (4.0%; *p* = 0.0089). However, both rates were higher than OA patients (0.65%) and controls (0.58%) (*p* < 0.0001).	Gonosome aneuploidy (X and Y) frequency were similar between KS and NOA patients (3.48% and 2.39%, respectively), but these rates were significantly higher than those patients with OA and controls (0.49% and 0.44%, respectively) (*p* < 0.0001). The same was true for chromosome 18 abnormalities frequencies (1.43%, 1.19%, 0.15%, 0.10% for KS, NOA, OA, and controls, respectively) (*p* < 0.0001).
[124]	Vozdova et al., 2012	Prospective	Testicular sperms from NOA patients (17)	Ejaculated sperms from normozoospermic donors (10)	Sperm numerical abnormalities for chromosomes X, Y, 13, 15, 16, 18, 21 and 22 by FISH	The frequency of disomy (2.32%) and diploidy (0.80%) was significantly higher in testicular sperm from men with NOA than in ejaculated sperm of normozoospermic donors (disomy: 0.62%; diploidy: 0.29%; *p* < 0.001 and *p* = 0.031, respectively).	NR
[125]	Weng et al., 2014	Retrospective	Embryos derived from 11 ICSI cycles of NOA patients by TESE (54) Embryos derived from 11 ICSI cycles of OA patients by MESA (58)	Embryos derived from 101 ICSI cycles of ejaculated sperm (460)	Embryo numerical abnormalities for chromosomes 8, 9, 13, 14, 15, 16, 17, 18, 20, 21, 22, X and Y	The rates of complex abnormalities were not statistically different between the MESA and TESE groups. The aneuploidy rate in each studied chromosome was not different among these three groups. The rate of abnormality in sex chromosomes did not differ from the rate of autosomal chromosomes, and there was no difference in the rates of abnormality between the X and the Y chromosomes.	There was a higher incidence of complex chromosomal abnormality in MESA-derived embryos than in TESE and ejaculated embryos.
[126]	Cheung et al., 2019	Prospective	Testicular sperms from NOA patients (4)	Epididymal sperms from OA patients (2) Ejaculated sperms from non-azoospermic infertile men (16)	Sperm numerical abnormalities for chromosomes 15, Y	Aneuploidy rates were higher in the ejaculated group (11.1%) compared to the epididymal sperm group from OA men (1.8%) and testicular sperm group from NOA men (1.5%) (*p* < 0.0001).	NR

FISH, fluorescence in situ hybridization; ICSI, intracytoplasmic sperm injection; IVF, conventional in vitro fertilization; KS, Klinefelter syndrome; MESA, microsurgical epidydimal sperm aspiration; NC, naturally conceived; NOA, nonobstructive azoospermia; NR, not reported; OA, obstructive azoospermia; OAT, oligoasthenoteratozoospermia; TESE, testicular sperm extraction.

**Table 4:** Studies evaluating psychological and neurodevelopmental features in ICSI offspring from NOA patients.

Reference #	Study	Design	Study Group (n)	Control Group (n)	Outcome Measures	Associations	
						NOA-ICSI Infants	NOA-ICSI Infants vs. Comparator
[140]	Tsai et al., 2011	Retrospective	TESE-ICSI infants (60) from NOA and OA patients	Children born after ICSI using freshly ejaculated sperm from men with severe OAT (21)	Perinatal outcomes and development of children assessed at the age of 1–7 years	No evidence of differences in the development of children after TESE-ICSI or ICSI using sperm from men with severe OAT.	No significant psychomotor or intellectual development delays were observed in all ICSI infants.
[136]	Tsai et al., 2015	Retrospective	ICSI infants (154); NOA (87)	OA (67)	Clinical outcomes	Children’s development outcomes were evaluated using a preschool developmental screening Table until 60 months of age. Items assessed: child’s feeding and sleeping behavior; posture; coordination; memory; problem-solving skills; language skills; socialization.	The general health of the children conceived using ICSI was satisfactory, with none showing major handicap in psychomotor or intellectual development.

ICSI, intracytoplasmic sperm injection; NOA, non-obstructive azoospermia; OA, obstructive azoospermia; OAT, oligoasthenoteratozoospermia; TESE, testicular sperm extraction.

## Data Availability

All data related to this manuscript are provided in the text.
